# Measuring Japanese mothers' perception of child abuse: development of a Japanese version of the child abuse blame scale – physical abuse (CABS-PA-J)

**DOI:** 10.1186/1751-0759-1-14

**Published:** 2007-07-10

**Authors:** Masaki Fujimoto, Taiko Hirose, Takeo Nakayama, Hiroji Okawa, Itsurou Takigawa

**Affiliations:** 1 Department of Nursing Function and Care Management, Tokyo Medical and Dental University Graduate School of Health Sciences, 1-5-45 Yushima, Bunkyo-ku, Tokyo 113-8549, Japan; 2 Department of Health Informatics, Kyoto University School of Public Health, Yoshida-Konoecho, Sakyo-ku, Kyoto 606-8501, Japan; 3 Okawa Children and Family Clinic, 1-6-16 Tamagawa, Ota-ku, Tokyo 146-0095, Japan; 4 Tokyo Metropolitan Ohtsuka Hospital, 2-8-1 Toshima-ku, Tokyo 170-8476, Japan

## Abstract

**Background:**

The Child Abuse Blame Scale – Physical Abuse (CABS-PA) was translated into Japanese and its subscale items modified by the authors according to the Japanese cultural context. The aim of the current study was to investigate the appropriateness, reliability, and clinical applicability of the CABS-PA Japanese version (CABS-PA-J). Modifications were made to enable the determination of child abuse recognition in a Japanese cultural setting and early clinical intervention in child abuse cases.

**Methods:**

The CABS-PA text was translated into Japanese, then back translated. The appropriateness of scale item translations was verified based on e-mail discussions with the original CABS-PA author. Exploratory and confirmatory factor analyses were performed to examine the validity of CABS-PA-J responses and to confirm the validity of factor structure. Criterion-related validity was also confirmed. The Japanese scale was used to examine the characteristic differences between mothers of premature infants (< 1500 g) and those of other infants (≧ 1500 g).

**Results:**

Exploratory and confirmatory factor analyses found the factor structure to be similar between the original scale and the translated CABS-PA-J, suggesting adequate factor validity. There was a statistically significant correlation between social support from a spouse or third party and the abuse score on a subscale, partially demonstrating criterion-referenced validity. Similarities and differences were found in the stress reactions of the mothers of premature infants (< 1500 g) and those of other infants (≧ 1500 g).

**Conclusion:**

CABS-PA-J was shown to be appropriate and reliable. It is an effective tool for determining the recognition of child abuse among Japanese mothers.

## Background

An increasing number of child abuse cases are being reported in Japan. Accordingly, social concern regarding child abuse has also grown over the last several years. Several contributing factors to this phenomenon have been pointed out: The number of nuclear families, who receive less sufficient support for childcare and thus may abuse children, has increased with changes in society [1] and society has become more aware of child abuse due to frequent media coverage of the problem, which may have resulted in increased criminal conduct [1,2].

Child abuse seldom occurs independently or with premeditation, rather it happens coincidently in relation to childrearing. Therefore, unraveling the causes of child abuse requires consideration of individual, family, community, and cultural influences, and how they interact dynamically during the critical formation of the parent-child relationship. The complexity of the parent-child relationship may make the prediction of child abuse difficult.

Interestingly, a certain pathognomonic attitude has been reported in abusive parents [3,4]. Dietrich *et al*. [5] discovered that abusive parents were more likely to justify their abusive behavior if they had been under considerable environmental stresses and thought that their children took a defiant attitude, while they were less likely to justify their abusive behavior if they recognized that they had lost their temper. Despite this, one limitation of the existing measures used to assess abusive parents is a failure to assess parental attitudes regarding the importance of child, parent, situational, and societal factors in motivating abuse [6].

Additionally, existing child abuse measures in Japan are not designed for use by non-abusive parents and childless adults. Thus, these groups are the most appropriate targets of child abuse prevention and educational programs. However, there is no Japanese scale for abusive parental attitudes that focuses on o prevention. Most child abuse scales focus on abusive behavior. Thus, these scales cannot be applied to prevention, but only for finding child abuse.

In Japan, research and early intervention in child abuse situations are performed by nurses, doctors, and psychologists. The Japanese Nursing Association has published statements on prevention, detection, and support for child abuse. Regardless, only a few community-based education programs with a multidisciplinary perspective that focus on nurses, the largest group of health providers, have been reported. It should be noted, therefore, that more than 200 nurse educators, clinicians and managers gathered for a one-day program designed to improve the understanding of the role of nurses, in order to combat the abuse and neglect of children and to formulate plans of action [7]. Moreover, pediatric nursing scientists and practitioners have published several reports on research and practice to promote healthy mother-child relationship development. For instance, the prenatal visit is one in which the nurse visits a pregnant woman's house to support her physical and mental health. They also provide group care for mothers who have child-care anxiety [8,9].

Moreover, when considered from a psychosomatic standpoint, the experience of child abuse continues to have a substantial influence on the individual's life, and many such cases present with psychosomatic symptoms. Accordingly, the influence of mental and physical injuries during childhood is an important topic in psychosomatic medicine, and primary and clinical research into the prevention and care of these injuries is a broad psychosomatic medical research category.

If the attitude of abusive parents can be measured, it might be useful for prevention. Petretic-Jackson [6] developed the Child Abuse Blame Scale – Physical Abuse (CABS-PA) to measure blame attribution for child abuse. This scale is a brief, easy-to-use screening measure to assess an important cognitive dimension of parental explanation and justification for abuse. Theoretically, attributions for blame play an important part in maintaining abusive behavior cycles and represent an important clinical target.

Therefore, the purpose of this study was to develop and refine the validity and reliability of a Japanese version of the Child Abuse Blame Scale – Physical Abuse (CABS-PA-J) in order to create tools for measuring the effects of child abuse prevention and educational programs for parents. We also intended to demonstrate the usefulness of the scale among mothers of infants, as well as to discuss the potential for practical use of the CABS-PA-J in the clinical setting.

In the first study, the validity and reliability of the CABS-PA-J was examined among mothers with children aged six years or less and among female undergraduates. In study 2, the utility of CABS-PA-J was examined when applied to the mothers of children three years of age who had normal birth weight and mothers with low-birth weight infants. This was done because the mothers of premature infants have been shown to have a theoretical risk factor of child abuse [10,11].

## Study 1: Translation, validity and reliability

### Subjects and methods

#### Translation into Japanese

The original scale, CABS-PA, was developed by Petretic-Jackson (PJ) [6]. It consists of a questionnaire that includes 30 items across four factors. These include the child's contribution, as misbehavior, to abuse; various environmental events that act as parental stressors; social acceptance of corporal punishment; and parental control of angry impulses. Respondents rate each item on the 6-point likert scale from "strongly disagree" to "strongly agree." Higher scores indicate a strong sense of placing blame on a particular factor.

After receiving permission from the original author (PJ), we translated the CABA-PA into Japanese, then back into English to confirm what we had done. The validity of these translated items was confirmed by the CABS-PA-J author. With the approval of the original author, we developed and added 13 original items reflecting each factor's concept, taking cultural differences into account.

#### Testing validity and reliability

We distributed anonymous self-administered questionnaires to 230 mothers of children six years of age or younger who went to a pediatric clinic in Ota Ward, Tokyo. We examined the validity of respondents' answers using the following two scales: the socially desirable scale (SDS) [12] and the lie scale (LS). We selected six items from the SDS and four from the LS. The SDS shows the tendency of respondents to give a socially desirable answer, and the LS items asked subjects about events that never happen in their actual life including "I am always healthy." For analysis, CABS-PA-J items were removed if they correlated significantly with SDS subscales. In addition, subjects who responded "yes" to all LS items were excluded.

We used exploratory and confirmatory factor analyses in order to assess factorial validity. We also calculated Pearson's correlation coefficients, comparing CABA-PA-J factors with the 10-item social support scale [13] developed by Tsutsumi and the child abuse behavior check list (CBCL) developed with reference to Seno [14], in order to test criterion-related validity. Previous studies indicated that abusive mothers were not socially supported by their relatives, and were socially isolated [15,16]. A scale for measuring social support from husbands and others was used in this study. The CBCL includes 18 items and asks mothers about abusive behavior that may have been committed, such as: "Have you slapped your child's head?" Subscales are classified into physical abuse, psychological abuse, and neglect. For examining internal consistency, Cronbach's alpha on each factor was calculated.

For assessing test-retest reproducibility, we recruited 49 female nursing undergraduates, ranging from 20 to 40 years-of-age, and asked them to answer the same CABS-PA-J questionnaire twice, at three-week intervals. Afterward, Pearson's correlation coefficients were calculated.

The study was approved by the Ethics Committee of Tokyo Medical and Dental University.

### Results

#### Exploratory factor analysis and Cronbach's alphas

The CABS-PA-J includes 43 items specific to the attribution of blame for child physical abuse. These items were subject to a maximum likelihood factor analysis with Promax rotation. The analysis produced a solution of four factors theoretically and based on possible interpretation. Each item required ≧ .35 of factor loading in order to be retained as a CABS-PA-J item. Items that overlapped significantly in content with other factor items were deleted. This process created a four-factor scale of 17 items. Although the new scale was organized somewhat differently from the original instrument, clear subscales were developed for Japan. These items had no significant correlation coefficients with SDS. Table 1 shows the four subscales and their contents. This resulted in a scale comprised of five “Child Victim,” four “Societal,” four “Situational,” four “Preserver-Perpetrator” items [see Additional file 1]. Taken together, the items emphasize either the child's role in contributing to the abuse by his or her misbehavior, the social acceptance of physical punishment, the various environmental events that act as parental stressors, and parental control of angry impulses. Cronbach's alphas obtained for these four categories were: .70, .73, .64, and .62 respectively.

#### Confirmatory factor analysis

**Figure 1:**
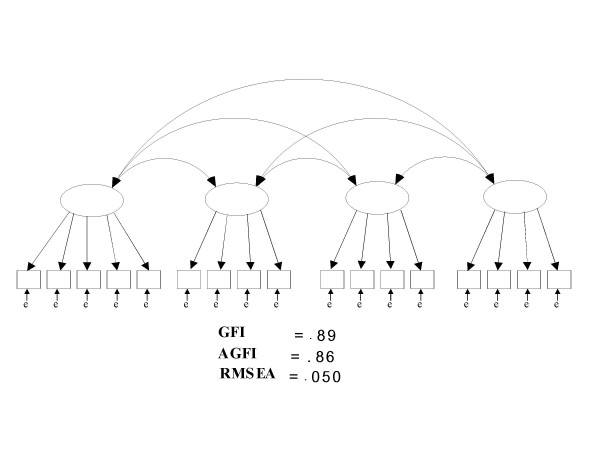
Oblique factor model.

To test whether CABS-PA-J would fit the factor analysis model, we conducted confirmatory factor analyses (CFA) for the four factors. Firstly, we examined the factors by CFA, which indicated that all factors fit each model. GFI and AGFI, goodness-of-fit indices of each factor, were acceptable (GFI = .97–1.00/AGFI = .91–.99). Secondly, we used CFA to examine 3 models: (1) orthogonal factor model, (2) oblique factor model, and (3) second-order factor model. The results revealed that the oblique factor model fits the CAS-PA-J better than the other factor analysis models. Table 2 shows that all goodness-of-fit indices of the oblique factor model, GFI, AGFI, and RMSEA were higher than the other models and that it was sufficient to be used as a model of CABS-PA-J [see Additional file 2]. Figure 1 is a graphical model of the oblique factor model. Thus, CABS-PA-J yields four sub-scores, with regard to the structure of the oblique factor model.

#### Criterion-related validity

The child victim score of CABS-PA-J correlated significantly with the social support by spouse (Pearson's r = -.27, N = 140, p < .01) and other persons (Pearson's r = -.27, N = 130, P < .01) (Table 3) [see Additional file 3]. The score also correlated significantly with total abusive score (Pearson's r = .20, N = 149, p < .05) and psychological abusive score (Pearson's r = .26, N = 151, p < .01); however, it only showed a trend with the physical abuse score (Pearson's r = .16, N = 149, p < .10). The total score of CABS-PA-J correlated significantly with the social support by spouse (Pearson's r = -.17, N = 140, p < .05), psychological abuse score (Pearson's r = .20, N = 150, p < .05), although only showing a trend with the total abusive score (Pearson's r = -.14, N = 149, p < .10). Other results were not significant. These results showed weak correlation among variables.

#### Test-retest reliability

The test-retest reliability of the CABS-PA-J subscales was examined. The child victim and societal scores after the three-week interval indicated high correlation with the initial results (Pearson's r = .82, N = 35, p < .01; Pearson's r = .70, N= 36, p < .01). The situational and preserver-perpetrator scores correlated to a mild degree (Pearson's r = .47, N = 36, P < .05; Pearson's r = .56, N = 36, P < .01).

## Study 2 Applicability and potential usefulness of the CABS-PA-J

### Methods

#### Subjects

**Figure 2:**
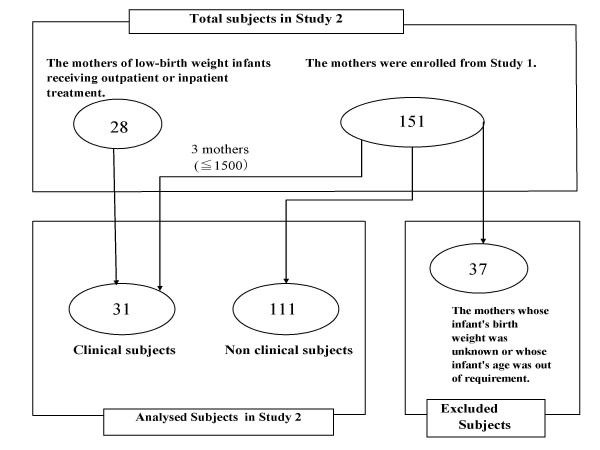
The subjects in study 2.

To investigate the applicability and potential usefulness of CABS-PA- J to Japanese mothers, study 2 was conducted as follows. Twenty-eight mothers of low-birth weight infants receiving outpatient or inpatient treatment in a hospital in Tokyo's Toshima Ward were enrolled into study 2 in addition to 114 subjects from study 1 (Figure. 2). Thirty-seven mothers whose infant's birth weight was unknown or whose infant's age was outside the criteria (1–3 years) were excluded. Of the study 1 subjects, only three were mothers of low-birth weight infants (≦ 2000 g: 1718 g, 1798 g, 1800 g).

In total, the 28 mothers of low-birth weight infants and three subjects with low-birth weight infants (≦ 2000 g) from study 1 were enrolled in study 2 as clinical subjects (Mean ± SD = 1015 ± 382 g). Of the clinical subjects, 22 infants were of very low birth weight (VLBW: ≦ 1500 g). The other 111 subjects were assigned as non-clinical subjects (Mean ± SD = 2975 ± 358 g). This is because 2000–2500 g low birth weight infants who do not have severe complications are usually saved without problem by contemporary medical technology.

Subjects were asked to complete the CABS-PA-J and the social support scale. Furthermore, we conducted two-way analysis of variance (ANOVA) on the CABS-PA-J subscales taking into consideration the infant's birth weight and age. We also obtained correlations between the CABS-PA-J subscales and the other factors for clinical and non-clinical subjects, using Pearson's correlation and Kendall's rank-correlation

## Results

### Birth weight and infant's Age

Firstly, two-way analysis of variance (ANOVA) was conducted on the CABS-PA-J subscales considering the infant's birth weight (greater or less than 2000 g) and infant age (greater or less than one year) (Table 4) [see Additional file 4]. ANOVA on the preserver-perpetrator factor showed the significant main effect to be from infant age (F (1, 133) = 8.93, p < .01). The results revealed that the mean preserver-perpetrator score of those mothers who had infants of one year of age or older was higher than that of those who had infants less than one year old. However, analyses of the other subscales produced no statistically significant results.

Secondly, two-way ANOVA was conducted on the CABS-PA-J subscales taking into consideration the infant's birth weight (greater or less than 2000 g) and infant age (greater or less than two years). ANOVA of the child victim factor showed the significant main effect to be from infant age (F (1, 131) = 9.56, p < .01). The results revealed that the mean child victim score of those mothers who had infants of two years or older was higher than that of those who had infants less than two years old. However, analyses of the other subscales produced no statistically significant results.

Thirdly, two-way ANOVA was conducted on the CABS-PA-J subscales taking into consideration the infant's birth weight (greater or less than 2000 g) and infant age (greater or less than three years). ANOVA of the Preserver-Perpetrator factor showed only a trend for Birth weight × infant age (F (1, 133) = 3.51, p < .10). However, analyses of the other subscales produced no statistically significant results.

Finally, we obtained correlations between the CABS-PA-J subscales and four factors (social support score [spouse or others], family income, mother's age, and mother's education level) for clinical and non-clinical subjects, using Pearson's correlation and Kendall's rank-correlation (Table 5) [see Additional file 5].

The higher preserver-perpetrator score of the clinical mothers was correlated with a lower degree of social support by others (Pearson's r = -.58, N = 23, p < .01) and mother's age (Pearson's r = .44, N = 29, P < .05). The child victim score showed only a trend with the mother's age (Pearson's r = .34, N = 28, p < .10).

For non-clinical mothers, the child victim score was significantly correlated with social support by spouse (Pearson's r = -.24, N = 98, p < .05) and by others (Pearson's r = -.41, N= 90, p < .01). The situational score was significantly correlated with the mother's educational level (Kendall's τ = .23, N = 107, p < .01). The preserver-perpetrator score showed a significant but weak correlation with the mother's education level (Kendall's τ = 0.17, N=107, p < .05). The societal score showed only a trend with the mother's age (Pearson's r = - 0.16,N=104, p < .10).

## Discussion

In the present study, we compiled the Japanese version (CABS-PA-J) of the self-report CABS-PA, developed by Petretic-Jackson [6], to measure the awareness of normal mothers and those with a tendency towards child abuse. The results of study 1 supported the sufficient validity and reliability of CABS-PA-J. In study 2, CABS-PA-J was carried out on mothers with low-birth weight infants (≦ 2000 g) up to age 3 (clinical group) and those with children up to age 3 weighing more than 2000 g at birth (non-clinical group) to investigate their characteristics. The results showed that both groups had similar tendencies as well as specific characteristics unique to the respective groups. These findings imply that CABS-PA-J is applicable for Japanese mothers and that it is potentially useful for revealing their cognitive characteristics toward child abuse.

### Review of CABS-PA-J reliability and validity

In study 1, we carried out a back translation when developing the scales to verify the validity of the contents. We also obtained the permission of the author of CAPS-PA, Petretic-Jackson, to modify scales that were adjusted to the Japanese population by adding new items, taking cultural differences into account.

Exploratory factor analysis produced four factors theoretically based on the criterion that the factor loading was above .35. Although these four factors differ slightly from the items in the original CABS-PA, their contents were essentially the same. However, the differences found from previous studies are believed to have been produced by differences in cultural background. In 2002, the number of calls made to child consultation centers around Japan asking for child abuse counseling increased to 24,000, a two-fold increase from 1999, and by 2004 had risen to 33,408, up 25.7% from 2003 [17,18] . It has only been over the last couple of years that Japanese society has actually started to demonstrate concerns over child abuse, which may explain the relative immature awareness of the Japanese towards abuse. Unlike the U.S., where countless studies on the subject have been conducted in the more than 100 years since the report of the 1874 Mary Ellen incident [19] and the study by Kempe [20] "Battered-Child Syndrome," the problem of child abuse is not adequately recognized in this country. Studies of child abuse awareness in Japan among professionals and general office employees have pointed out that adult persons with general office jobs tend to have less awareness of psychological abuse compared to those working in the areas of education and childcare, that awareness of abuse in the country is diverse, and that raising common awareness and understanding involves numerous difficulties [21-24]. Next, results of reviewing alpha reliability coefficient and test-retest reliability of the scale confirmed that CABS-PA-J is generally reliable. The child victim and societal factors were also found to serve as independent scales due to their high reliability.

In confirmatory factor analysis, the oblique factor model was found to be the most suited to CABS-PA-J of the three models. In addition, GFI, AGFI, and RMSEA, the goodness-of-fit indices of the oblique factor model, also indicated sufficient compatibility with CABS-PA-J. In the present studies, GFI was .89, indicating sufficient compatibility. RMSEA below .050 indicates models with sufficient goodness of fit, and RMSEA below .080 was also taken to be suitable, therefore, .050 was considered sufficient [25]. Consequently, the results of the analysis of confirmatory factors in the present studies verified that goodness-of-fit indices were excellent, and that structural concept validity was also sufficiently appropriate.

The results of criterion-related validity examination showed a significant negative correlation comparing the CABS-PA-J subscale child victim with social support by spouses and by others. In addition, the subscale was found to have a significant trend with the physical abuse score, significant positive correlation with psychological abuse score, as well as significant correlation with the child abuse behavior checklist total score. The total CABS-PA-J score, on the other hand, had a significant negative correlation with social support by spouse, positive correlation with the abuse behavior checklist subscale "psychological abuse," and a significant positive correlation with the child abuse behavior checklist total score. The four CABS-PA-J subscales were weak in relation to other scales in existence, because the concepts of existing scales were for other parameters.

Consequently, the criterion-related validity of CABS-PA-J has been demonstrated to a certain extent. These results also suggest, as pointed out by the belief theory proposed by Wright *et al*. [26] and theories of cognitive behavioral therapy [27], that cognitive variables play an important role in factors influencing interactions between people, which may be evident theoretically. The results of the present studies show that CABS-PA-J is an appropriate scale for measuring the mother's awareness and belief of abuse, and suggest that, as a result of that awareness, a cognitive variable may be involved in abusive acts towards their children.

However, such awareness (belief) can change according to the mother's situation [28]. Aramaki demonstrated that if social support by the husband is sought but not acquired by the mother, her positive feelings towards their child are the weakest [29]. The results of the present studies also confirmed a significant negative correlation between the CABS-PA-J subscale and high social support, as well as between the subscale and the child victim factor. This suggests the presence of a process where the stressful situation of not receiving social support interacts with awareness of abuse, resulting in abusive acts.

Although the original scale targets awareness towards physical abuse, the CABS-PA-J showed a higher association with the psychological than the physical abuse scores. These results should be interpreted taking into consideration the following points: the present studies were conducted of mothers who took their children to our hospital; and, since there were no mothers who were actually abusing their children physically, CABS-PA-J may have demonstrated an association with psychological abuse. However, the premise of this study involved a model whereby a one-dimensional correlation exists for abusive parents as well as those that do not abuse their children. In addition, as this was primary research aimed at developing a scale, the subjects weren't limited to those parents who committed child abuse. Consequently, we consider the results of this study to be valid.

The results of a review of the criterion-related validity found that the CABS-PA-J subscale is associated with social support and abusive acts, and that the CABS-PA-J total score shows the same tendency. Therefore criterion-related validity is thought to have been demonstrated to a certain extent.

### Characteristics found by CABS-PA-J: Mothers of low-birth weight children up to age 3 (clinical group) vs. Mothers of standard-birth weight children (standard Group)

The purpose of Study 2 was to investigate whether or not CABS-PA- J can be clinically applied. Mothers of low birth weight children up to age 3 (clinical group) and those of standard birth weight children (standard group) were compared using the CABS-PA-J subscales. The results revealed that mothers of children over one year had higher preserver-perpetrator scores than those of children less than one year. The preserver-perpetrator is thought to be a factor indicating guilty feelings by the parent based on the view that the parent is responsible for abuse, with the score being higher at about age one [30], when attachment to infants becomes stable. In general parent-child relationships, therefore, if child abuse occurs when the parent's attachment to the child is established, then the mother usually recognizes that she is wrong.

ANOVA of the two factors, age (< 2 years and ≧ 2 years) and birth weight, found the main effects of age in term of the CABS-PA-J child victim. The child victim score increased in mothers of infants aged over two years. When a child reaches age two, his/her range of actions broadens, and the mother experiences more stress and anxiety in bringing up her child, which may be why the child victim score increases [31,32].

Essentially, ANOVA of the two factors, age (< 3 years and ≧ 3 years) and birth weight found the only a trend in terms of the CABS-PA-J Preserver-perpetrator.

In addition, correlation analysis comparing CABS-PA-J subscales with respective social support, family income, mother's age, and mother's education level demonstrated a difference in the characteristics of the two groups: mothers of low-birth weight children up to age three (clinical group) and those of standard-birth weight children (standard group).

Mothers in the clinical group showed a significant negative correlation between the CABS-PA-J preserver-perpetrator subscale and support by others and a significant positive correlation between the subscale and mother's age. This indicates that the higher the mother's age, the higher the preserver-perpetrator tendency.

On the other hand, mothers in the standard group showed a significant negative correlation between the child victim subscale score and any social support by spouse or support by others. In other words, social support to mothers in the standard group showed a significant negative correlation with child victims. This consequently suggests that increasing social support may help reduce abusive acts in the standard group. There was also a significant positive correlation between the situational factor and the mother's education level. Situational factors, attributing child abuse to the situation, consist of items including various environmental stressors to the parents [6]. Subsequently, it shows the possibility that even mothers in the standard group, who have no concerns about the physical development of their child, may feel environmental stress more easily if they have a higher educational background. Moreover, mothers in the standard group showed significant correlations with the preserver- perpetrator and mother's education level; specifically, mothers with high educational background are more likely to think that they are responsible for child abuse, which serves as a strong control over child abuse.

In this way, the results of calculating the correlation between CABS-PA-J subscales and respective social support, family incomes, mother's age, and mother's education level between the clinical and standard groups indicated characteristic differences in the correlation pattern by parameter. Mothers from the clinical group showed a strong tendency to have guilty feelings [33,34]. Since it has been pointed out that giving birth during middle age poses as an environmental factor of low-birth weight children [35], mothers will tend to feel a greater sense of guilt towards their child, represented by the preserver-perpetrator scale, and as age increases. However, since the subscale showed a significant negative correlation with social support by others, increased social support by others may help reduce preserver-perpetrator scores in such cases.

As a result of a review of the differences in correlation patterns between the two groups, awareness of child abuse was found to have similar progress as child development between the two groups. On the other hand, the significance of their awareness may be dissimilar. In other words, even if no substantial difference is seen in development by age, the mother's feeling of guilt will increase and she may develop depression as a result if her age is high, as in the present studies, and the child is born at a low birth weight. Breslau and Davis [36] reported that 30% of middle-aged mothers who gave birth to babies with low birth weight suffer serious depression. In addition, their daily stressor was associated with a lack of social resources. These can be predictors of both the severe state of depression in mothers as well as their potential for child abuse [37]. A number of studies have shown that most abusive mothers lack social support as well as social resources and suffer from severe depression [37-40]. Therefore, the results of the present studies suggest that mothers of low-birth weight children have higher risks of developing depression, and thus higher risks of abusing their children.

Singer *et al*. [41] reported that mothers of extremely-low birth weight children who do not have high risks experience the same psychological pain as mothers of term infants at the point their children become two years old. Even though the parenting stress of mothers of high-risk, low-birth weight children continues to increase, several psychological adaptation scales show the same scores as mothers of term infants when their children become three years of age. On the other hand, mothers of high-risk, low-birth weight children show positive adaptation on the whole at three years, yet they are still reported to suffer from five times more serious psychological pain than mothers of term infants; almost one-third of mothers of ultra-low birth weight children are reported to suffer depression and anxiety at the clinical level. The results of this study also suggest that the older the mothers of low birth weight children, the greater their guilt feeling will be towards their children, resulting in psychological pain.

Since social support by others correlated negatively with the preserver-perpetrator phenomenon, it also suggests that complete support may be important to such mothers, indicating the importance of support by professionals to such children and their families.

### Limitations of this study and the clinical use of CABS-PA-J in the future

The results of this study showed that CABS-PA-J has moderate validity and reliability as a scale for measuring awareness towards child abuse. It also successfully clarified several cognitive characteristics of mothers of the two study groups. Because the subjects of the present studies were mothers and children who visited hospitals, however, the results may include sampling bias instead of actual abuse. The potential of a difference in awareness towards abuse between the two groups of mothers was also hinted at. In the future, to enable use of this scale in the actual clinical setting, there will be a need to continue studies of mothers who are actually abusing their children, as well as studies of association with other psychological processes.

On the other hand, the results suggested an association between awareness of child abuse and actual acts. The combination of the CABS-PA-J scale and other scales for measuring environmental stressors of parents may help predict the risks of child abuse without having to actually ask mothers directly if they are abusing their children, which may help prevent child abuse from an early stage. Moreover, there is the potential to create educational programs for parents who commit child abuse, using CABS-PA-J to specify the educational material needed for those parents and to create individual training programs for them.

Furthermore, the use of the scale for mothers receiving therapy for child abuse may help determine changes in attitude and assess therapeutic effects. In order to enhance clinical efficacy in the future, further studies using the CABS-PA-J should be carried out.

## Authors' contributions

MF had the original idea for the survey and all aspects of the study, TH and TN contributed to the design of the study, and analysis and interpretation of data, IT and HO contributed to the acquisition of data. All authors read and approved the final version of the paper.

## Supplementary Material

Additional file 1 Table 1, Factors and items of CABS-PA-J. Click here for file

Additional file 2 Table 2, Confirmatory factor analysis of CABS-PA-J. Click here for file

Additional file 3 Table 3, Correlation between CABS-PA-J subscales and social support or abusive behavior. Click here for file

Additional file 4 Table 4- 1 to 3, ANOVA of age and birth weight of children according to CABS-PA-J factors. Click here for file

Additional file 5 Table 5, Correlations between CABS-PA-J subscales and social support, family income, or education level in mothers of children up to age 3. Click here for file

## References

[B1] Takahashi S (2001). Child Abuse: The worst violation of children's rights by parents.

[B2] Ueno K, Nomura T (2003). The establishment of child abuse : The family in captivity.

[B3] Milner JS (1993). Social information processing and physical child abuse. Clin Psychol Rev.

[B4] Milner JS, Hansen DJ (2000). Social information processing and child physical abuse:Theory and research. Nebraska Symposium on Motivation Motivation and child maltreatment.

[B5] Dietrich D, Berkowitz L, Kadushin A, McGloin J (1990). Some factors influencing abusers' justification of their child abuse. Child Abuse Negl.

[B6] Petretic-Jackson P, VandeCreek L, Jackson T, Knapp S (1992). The Child Abuse Blame Scale – Physical Abuse: Assessing blame for child physical abuse. Innovations in Clinical Practice.

[B7] Henry BM, Ueda R, Shinjo M, Yoshikawa C (2003). Health education for nurses in Japan to combat child abuse. Nurs Health Sci.

[B8] Niitsu N (2003). An effective method of resolving childrearing anxiety, confuse: Prenatal visit. J Child Health.

[B9] Matsunogo Y, Mizuno M, Aida I, Takei A (2004). Group psychotherapy for women with difficulties in mothering. J Child Health.

[B10] Friedrich WN, Boroskin JA (1976). The role of the child in abuse: a review of the literature. Am J Orthopsychiatry.

[B11] Wu SS, Ma CX, Carter RL, Ariet M, Feaver EA, Resnick MB, Roth J (2004). Risk factors for infant maltreatment: a population-based study. Child Abuse Negl.

[B12] Crowne DP, Strickland BR (1960). A new scale of social desirability independent of psychopathology. J Consult Psychol.

[B13] Tsutsumi A, Kayaba K, Ishikawa S, Kario K, Matsuo H, Takuma S (2000). Jichi Medical School Social Support Scale(JSS-SSS) revision and test for validity and reliability. Nippon koshu eisei zasshi.

[B14] Seno E (2002). Child Abuse: Present situation deemed serious. Jpn J Child Abuse Negl.

[B15] Gray JD, Cutler CA, Dean JG, Kempe CH (1977). Predication and prevention of abuse and neglect. Child Abuse Negl.

[B16] Grietens H, Geeraert L, Hellinckx W (2004). A scale for home visiting nurses to identify risks of physical abuse and neglect among mothers with newborn infants. Child Abuse Negl.

[B17] Ministry of Health and Labour and Welfare (2004). a: White Paper on Health, Labour and Welfare.

[B18] Ministry of Health and Labour and Welfare (2004). b: Brief summary of 2004 Annual Report on Social Welfare service.

[B19] Child Abuse Prevention Workshop (1993). Preventing Child abuse.

[B20] Kempe CH, Silverman FN, Steel BF, Droegemueller S, Silver HK (1962). The battered-child syndrome. JAMA.

[B21] Japanese Nurse Association (2003). Nurse's Hand book of preventing child abuse and caring for abused child.

[B22] Sato Y, Endo K, Shiwaku H, Yamato Y (2003). Attitude toward child abuse of high school students. Yamagata J Health Sci.

[B23] Miwa M, Iwashimizu T, Suzuki F, Yamaya H (2004). Perception of Maltreatment of Child among Public Health Nurses. Journal of Nursing, Shiga University of Medical Science.

[B24] Ninomi K, Shinohara Y, Fujita M, Tsuda A, Nishimura M, Seki H (2004). A study on The factors concerning the recognition of child abuse and neglect using multiple logistic regression analysis. J Child Health.

[B25] Arbuckle JL, Wothke W (1999). Amos 40 User's Guide Small Waters Corporation.

[B26] Wright LM, Watson WL, Bell BeliefsJM (1996). The heart of healing in families and illness.

[B27] Beck AT, Rush AJ, Shaw BF, Emery G (1979). Cognitive therapy of depression.

[B28] Shimizu Y (2003). The relationship between Maternal Belief of Motherhood and Their Childcare-related Stress. J Child Health.

[B29] Aramaki M (2005). The relationships among child-rearing anxiety, affirmative feeling for child rearing, and social support: A comparison between single and married mothers. J Child Health.

[B30] Bowlby J (1982). Attachment and loss.

[B31] Kawasaki H, Kaihara Y, Kosaka S, Deji A, Katano T (2005). Relationship between anxieties of mothers on child rearing and their feelings on family function. J Child Health.

[B32] Kawai H, Shoji J, Chiga Y, Kato H, Nakano E, Tsunetsugu K (1995). A clinical study on matermal anxiety related to child rearing("Ikuji-Fuan"). JCFRI Bulletin.

[B33] Morikawa H, Deguchi Y, Kusaka Y, Takeuchi T, Nakagawa Y, Satake N (2000). Follow-up study related to raising Low-Birth-Weight Infants.: Relationship amog physical growth, mental development and child-rearing anxiety. Jpn J of Public Health.

[B34] Nagata M (2002). An early intervention for mother and premature infants. : The therapeutic process of mother infant psychotherapy : A case study of a mother with difficulties in ELBWI rearing. J Jpn Clin Psychol.

[B35] Nanbu H (2001). Low birth weight infant. Jpn J of Pediatrics.

[B36] Breslau N, Davis GC (1986). Chronic stress and major depression. Arch Gen Psychiatry.

[B37] Sachs B, Hall LA, Lutenbacher M, Rayens MK (1999). Potential for abusive parenting by rural mothers with low-birth-weight children. Image J Nurs Sch.

[B38] Spinetta JJ, Rigler D (1972). Parental personality factors in child abuse. J Consult Clin Psychol.

[B39] Chaffin M, Kelleher K, Hollenberg J (1996). Onset of physical abuse and neglect: psychiatric, substance abuse, and social risk factors from prospective community data. Child Abuse Negl.

[B40] Saito S (2003). The battering parents: Their characteristics and pathologies. Jpn J of Child Abuse Negl.

[B41] Singer LT, Salvator A, Guo S, Collin M, Lilien L, Baley J (1999). Maternal psychological distress and parenting stress after the birth of a very low – birth – weight infant. JAMA.

